# Importance of Assessing Muscular Fitness in Secondary Care

**DOI:** 10.3389/fgene.2020.583810

**Published:** 2020-10-28

**Authors:** Barbara Strasser

**Affiliations:** Medical Faculty, Sigmund Freud Private University, Vienna, Austria

**Keywords:** sarcopenia, muscle assessment, physical performance, exercise, older people, secondary care

## Introduction

Muscle atrophy is an unfortunate effect of aging and many diseases and can compromise physical function and impair vital metabolic processes (Argilés et al., 2016; Strasser et al., 2018). One of the many threats to independent life is the age-related loss of muscle mass and strength (by ~1% per year from the age of around 30 years) referred to as sarcopenia (Goodpaster et al., 2006). These structural and functional changes in skeletal muscle contribute significantly to adverse health outcomes such as falls, fractures, functional impairments, and mobility limitations accompanied by elevated risk for hospitalization, morbidity and mortality in older persons (Bianchi et al., 2016). Furthermore, malnutrition is common among older people and often poorly recognized and underdiagnosed (Roberts et al., 2019). Insufficient dietary intake is not only related to the development of sarcopenia (Beaudart et al., 2019), but is also a major risk factor for cognitive or functional impairments and mortality in older patients (Sánchez-Rodríguez et al., 2019; Zanetti et al., 2019).

In a recent systematic review with data of a total of 34,955 participants older than 60 years, the prevalence of sarcopenia in community-dwelling individuals was 11% in men and 9% in women, whereas in nursing-home individuals the prevalence was 51% in men and 31% in women and in hospitalized individuals 23 and 24% for men and women, respectively (Papadopoulou et al., 2020). Nevertheless, health care practitioners often inadequately address the multifactorial issues that contribute to age-related and disease-related skeletal muscle changes, such as the following key factors: reduced physical activity and/or energy intake, anabolic resistance, changes in hormones (mainly sexual hormones, growth hormone, insulin-like growth factor 1, and insulin), and low-grade systemic inflammation (Cruz-Jentoft et al., 2010). Sex-specific hormonal changes that occur with aging, with reduced amounts of testosterone and estrogen in men and women, respectively, are an important factor related to sex differences in skeletal muscle structure, function, and metabolism (Gheller et al., 2016), and furthermore, a major contributing factor to the development of sarcopenic obesity with age, associated with accelerated functional decline and increased risks of cardiometabolic diseases, compared to sarcopenia or obesity alone (Roh and Choi, 2020).

The purpose of this opinion article is to reinforce the necessity of easy-to-use clinical measures for identifying patients at risk of developing sarcopenia or related disorders and to further provide practical guidelines that should be considered in the implementation of an exercise program in secondary care.

## Measurement of Muscular Fitness in Clinical Settings

As an objective measure, the assessment of muscular strength has achieved considerable clinical value and is considered as a key characteristic of sarcopenia with low handgrip strength (<27 kg for men and <16 kg for women) as the first defining characteristic (Cruz-Jentoft et al., 2018). A recent meta-analysis evaluating the relationship between muscular strength measures and mortality in outpatient populations with chronic diseases and in critically ill hospitalized patients found evidence of associations between all measures of muscular strength investigated (handgrip strength, knee extension strength, and other) and all-cause mortality (Jochem et al., 2019). This growing body of evidence should be a stimulus for physicians to incorporate muscular strength improvement as a high priority in the overall clinical treatment approach to patients with multiple chronic conditions to improve patient management and patient health, though it may be even better to use regular exercise as a preventative strategy. Being physically active is one of the most important approaches that people of all ages can take to maintain their health and normal functioning. Future trials should be conducted to develop validated cut-points for diagnosing low muscular fitness and their predictive value for hard end-points, such as survival.

The implementation of simple physical capability tests that may reliably assess the muscular strength state of the patient has value in any phase of aging, but particularly in the elderly routine because of the bidirectional interplay between multimorbidity and functional impairment (Calderón-Larrañaga et al., 2019). Recent findings of a prospective study from UK Biobank suggested that from all physical capability markers used to define sarcopenia, slow gait speed (≤0.8 m/s) and low handgrip strength seemed to be the main drivers of the noticed association with health outcomes—more than low muscle mass—and should be considered in clinical practice (Petermann-Rocha et al., 2020). According to the concept of “coordinated deadaptation,” functional capacities (and structure) of the cardiovascular and respiratory systems will decline when skeletal muscle function due to aging and disease, but also as a result of physical inactivity, decreases (Burtscher, 2013). The Longevity Check-up 7+ project recently showed that pulmonary function was positively associated with muscular function, assessed by chair stand and handgrip strength tests (Landi et al., 2020). Thus, timely detection of lower muscular strength and function may be helpful in evaluating potential pulmonary function impairment. This loss of function is often the result of changes in muscle quality independent of muscle mass (Correa-de-Araujo et al., 2017). Potential mechanisms include changes in muscle tissue composition and muscle cell metabolism based on high levels of inter- and intra-muscular adipose tissue and intramyocellular lipids. For future clinical practice, measures of muscular strength can be combined with measures of muscle quality such as phase angle (Uemura et al., 2020) or ultrasound-measured thigh muscle echogenicity, also known as echo intensity, to create a score that would better predict functional strength and clinical outcomes across the adult age span (Bourgeois et al., 2019). Although more expensive and time-consuming, the use of magnetic resonance imaging (MRI) allows additional evaluation of fat/connective tissue infiltration of the muscles (Prado and Heymsfield, 2014). This enables a more tailored approach for treatment, which may help in improving the effectiveness and acceptability of therapies currently available.

## Recommendations for the Management of Sarcopenia

Initiating early treatments to maintain proper muscle mass and function are crucial for optimal patient outcomes across the healthcare continuum (Prado et al., 2018). Interventions to support muscularity include resistance exercise and nutrition because both have a positive impact on protein anabolism. An initial resistance training program should be performed on 2 non-consecutive days per week and may progress to a regimen of 3 days per week. The training load should be systematically increased to keep the maximum possible repetitions per set between 10 and 15, corresponding to 60–80% of one- repetition maximum (Fragala et al., 2019). A minimum of two sets per muscle group per week should be performed at the beginning of the program and be increased progressively (every 4 weeks by one set) to a maximum of six sets (in rehabilitation) and 10 sets (in health promotion) per muscle group per week (Strasser and Schobersberger, 2011). Generally, resistance training should consist of exercises for all major muscle groups. However, training of the small muscle groups of the lower limbs (e.g., single leg knee extensions) is a powerful approach to combat exercise intolerance in patients with chronic obstructive pulmonary disease and heart failure (Burtscher, 2013). Although traditional slow-velocity resistance training is primarily associated with improvements in muscular strength level, there is convincing evidence that muscle power training with higher-velocity and lower-intensity (30–60% of one repetition maximum or the use of own body as resistance) would be a more effective strategy to improve both muscular strength and power output, as well as functional abilities (i.e., sit-to-stand, walking ability, stairs climbing) in elderly populations, including the frail oldest old (Cadore and Izquierdo, 2018).

However, poor exercise compliance and adherence to exercise training programs is a common problem in older multimorbid patients. In these cases, health care professionals should focus on patients' strengths rather than their weaknesses. Motivating patients to be active can help alleviate the loss in muscular function associated with aging and disease. The clinical environment can be easily used for health promotion activities, in particular advocating for reduced sedentary time and increased physical activity to promote muscle conditioning and thereby supporting patients' self-management (Murayama et al., 2020). Nevertheless, many of the barriers to exercise and some of the reasons for poor adherence come from outside the clinical environment, such as lack of time or lack of skills, costs of sports programs or equipment. With time economy as the primary concern, the 2018 US Physical Activity Guidelines for Americans revealed new opportunities for promoting physical activity by recognizing that even short and sporadic bouts of high relative intensity incidental physical activity count for health (Piercy et al., 2018), which may be an attractive option for people living a sedentary lifestyle to be more active from earlier in life to stop them having problems later on.

For patients who are not able to perform active exercise, such as in the ICU setting, the use of neuromuscular electrical stimulation (NMES) appears to be a potential adjunct to prevent muscle atrophy and loss of muscle strength (Dirks et al., 2015). Moreover, this technique is a useful clinical tool to preserve leg lean mass during hospitalization in geriatric patients (Karlsen et al., 2020). At the molecular level, NMES stimulates the regenerative capacity of satellite cells and induces downregulation of genes (e.g., myostatin, MuRF1 and MAFbx) involved in muscle atrophy (Karlsen et al., 2020). NMES intensity should be as high as individually tolerated, and a minimum of three sessions per week with large pulses (between 300 and 450 μs) and high frequency (50–100 Hz in young and around 30 Hz in older adults) should be performed (Adams, 2018).

A recent RCT in elderly men found that whey protein supplementation following resistance exercise induced changes in muscle microRNAs (miR-208a and-499a and-206) similar to what is reported in young men (D'Souza et al., 2019). These findings confirm a potential involvement of specific microRNAs in the regulation of hypertrophic signaling pathways following an acute resistance training stimulus. Thus, circulating microRNAs may serve as a predictive marker of the physiological state of skeletal muscle and may have important significance for the screening of early sarcopenia and related conditions (He et al., 2020), but also provide an understanding into mechanisms involved in the aging process such as anabolic resistance (Margolis et al., 2017).

In addition, the supplementation of essential amino acids and vitamin D can further augment protein anabolism and has been shown to improve muscle composition in mobility-limited older adults (Englund et al., 2017). The current recommended dietary allowance (RDA) for protein of 0.8 grams protein per kilogram of body weight per day might not be adequate for maintaining muscle health in old age. Therefore, experts have proposed an increase in dietary protein recommendations for older age groups to 1.0–1.2 g/kg body weight per day, and an even higher protein intake (1.2–1.5 g/kg body weight/day) is advised for those who are exercising or for older people with disease or injury (Bauer et al., 2013). Nevertheless, the training component *per se* is of primary importance when it comes to improving muscle mass and strength, as well as functional capacity, as a substantial part of the older population does benefit from a resistance-type exercise training intervention (Churchward-Venne et al., 2015).

Although testosterone supplementation in older men has been shown to improve body composition, yet the effects on muscular strength or physical function are still conflicting (Endo et al., 2020). It has been shown that testosterone replacement therapy does not offer any benefit beyond resistance exercise alone in elderly patients with low to normal serum testosterone, but may be an effective short-term intervention to overcome age-related deficits in adaptive responses to resistance training (Gharahdaghi et al., 2019). However, because of potential adverse events, the clinical meaningfulness of testosterone in the management of sarcopenia should be carefully evaluated.

**Figure 1:**
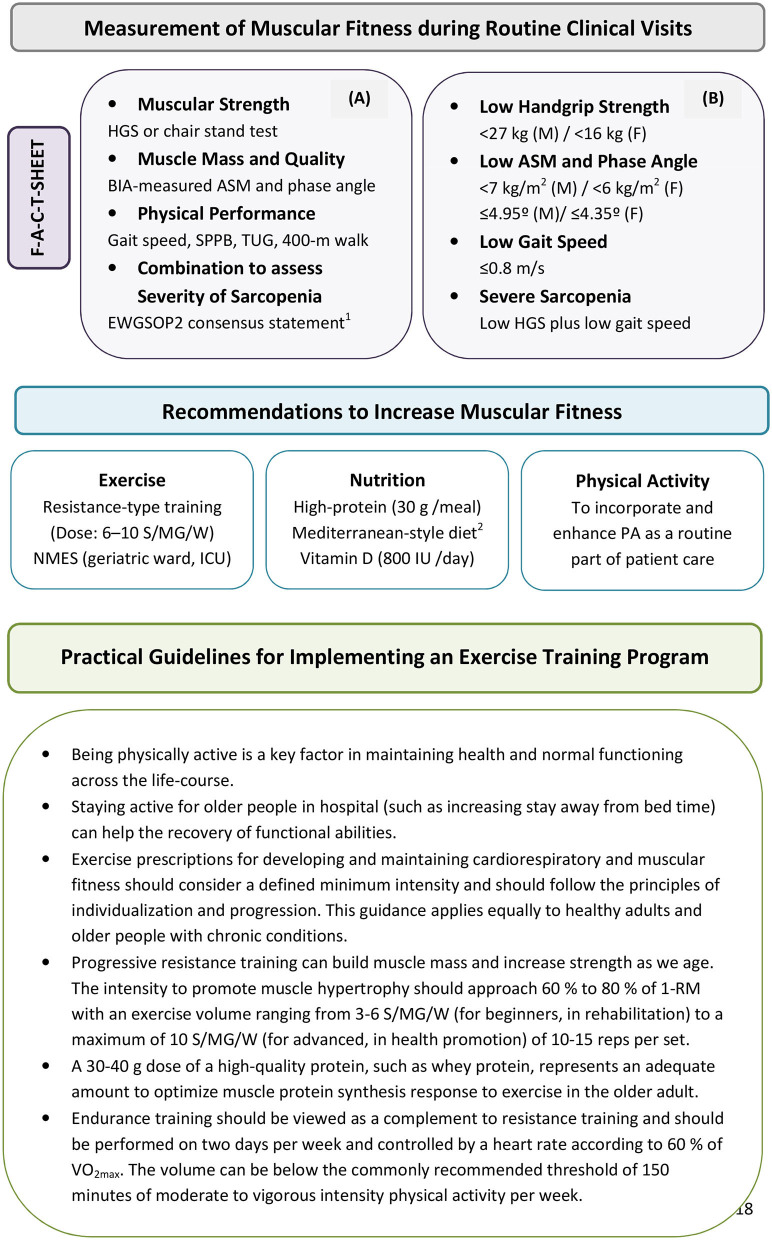
Procedures for incorporating muscular strength and function estimations into routine clinical assessments in a pragmatic manner **(A)** for identifying patients at risk for low muscular fitness in secondary care. **(B)** Recommendations listed in figure provide practical guidelines that should be considered in the implementation of an exercise and physical activity program. The steps of the pathway are represented as Find-Assess-Combine-Treat or F-A-C-T [modified by Cruz-Jentoft et al. (2018)]. EWGSOP2 recommends for case-finding the use of the SARC-F questionnaire as a screen for sarcopenia risk (Malmstrom et al., 2016). ^1^Cruz-Jentoft et al. (2018); ^2^Capurso et al. (2019). ASM, appendicular skeletal muscle mass; BIA, bioelectrical impedance analysis; EWGSOP2, European Working Group on Sarcopenia in Older People 2; F, females; HGS, handgrip strength; ICU, intensive care unit; M, males; MD, mediterranean diet; NMES, neuromuscular electrical stimulation; PA, physical activity; S/MG/W, sets per muscle group per week; SPPB, Short physical performance battery; TUG, Timed-up-and-go test; 1-RM, one-repetition maximum; VO_2max_, peak oxygen uptake.

It is essential to maintain good muscular fitness over the long-term to improve health outcomes. In this regard, it is important to incorporate physical performance assessment and promotion into healthcare in a manner that engages both clinicians and patients (Brannan et al., 2019). Accordingly, understanding and addressing muscular strength, mass, and function in older persons and to communicate the health-promoting effects of regular exercise and physical activity combined with nutritional advice affords clinicians with a vitally important opportunity to identify patients at risk of developing sarcopenia or related disorders and, more importantly, to preserve muscle mass and function. Procedures for incorporating diagnostic tools into routine clinical practice in a pragmatic manner and recommendations that should be considered in the implementation of an exercise training program to increase muscular fitness are provided in Figure 1. Such a multimodal approach improves the efficiency of therapy and ameliorates the functional capacity even in advanced disease stages; these are crucial to avoid long-term care, thereby promoting quality of life for older people.

## Author Contributions

The author confirms being the sole contributor of this work and has approved it for publication.

## Conflict of Interest

The author declares that the research was conducted in the absence of any commercial or financial relationships that could be construed as a potential conflict of interest.
